# How to repair medial meniscal ramp lesions: A systematic review of surgical techniques

**DOI:** 10.1002/jeo2.12037

**Published:** 2024-06-17

**Authors:** Nicola Pizza, Luis L. Urda, Francisco S. Sanchez, Maximiliano Ibañez, Stefano Zaffagnini, Simone Perelli, Juan C. Monllau

**Affiliations:** ^1^ Knee and Arthroscopy Unit, ICATME, Hospital Universitari Dexeus Universitat Autònoma de Barcelona Barcelona Spain; ^2^ Clinica Ortopedica e Traumatologica II IRCCS Istituto Ortopedico Rizzoli Bologna Italy

**Keywords:** medial meniscus, meniscus repair, ramp lesion

## Abstract

**Purpose:**

to provide a comprehensive overview of all the surgical techniques published in the literature for repairing meniscal ramp lesions focusing on the technical aspects and the pros and cons of every procedure. Such lesions can be managed using various approaches, each of this with its specific advantages and disadvantages.

**Methods:**

Pubmed Central, Scopus, and EMBASE databases were systematically reviewed according to the preferred reporting items for systematic reviews and meta‐analysis (PRISMA) guidelines for studies on surgical techniques for repairing meniscal ramp lesions through May 2023. Overall, 32 articles matched the selection criteria and were included in the study.

**Results:**

Debridement alone may be sufficient for small stable meniscal ramp lesions but, for tears in the menisco‐capsular junction that affect the stability of the medial meniscus, it seems reasonable to repair it, even though the clinical results available in literature are contrasting. All‐inside sutures through anterior portals seems to be an effective solution for meniscal ramp lesions with MTL tears. All‐inside sutures through posteromedial portals are particularly useful for large meniscal ramp lesions, in which an inside‐out suture can also be performed.

**Conclusion:**

Meniscal ramp lesions can be managed using various approaches, each of this with its specific advantages and disadvantages. Further research is required to determine the optimal technique that can be considered as the gold standard and can provide the better results.

**Level of Evidence:**

Level III, systematic review.

**Abbreviations**
ACL: anterior cruciate ligament
MCL: medial collateral ligament
MRI: magnetic resonance imaging
MTL: meniscotibial ligament
PCL: posterior cruciate ligament
PHMM: posterior horn medial meniscus
PRISMA: preferred reporting items for systematic reviews and meta‐analysis

## INTRODUCTION

Meniscal ramp lesions have gained a renewed interest due to its high correlation with anterior cruciate ligament (ACL) tears, up to 42% [7, 34, 40], and when this occurs there is an even greater increase of the anterior tibial translations and rotational laxity, and it has been shown that such biomechanical modifications could drive to increased cartilage degeneration if left unrepaired [19]. A classification system has been proposed for meniscal ramp lesions, with five initial types based on tear location: type 1 involves meniscocapsular tears located peripherally in the synovial sheath; type 2 comprises partial superior lesions; type 3 corresponds to partial inferior lesions; type 4 involves complete tears in the red‐red zone; and type 5 corresponds to double tears [42]. This classification has been subsequently modified with the addition of two subtypes for type 3 and type 4 lesions: type 3A represents partial inferior lesions with an intact meniscotibial ligament (MTL), while type 3B represents partial inferior lesions with a MTL tear; type 4A involves complete tears in the red‐red zone with an intact meniscocapsular attachment but connected to the free‐floating fragment of the PHMM; and type 4B involves a complete tear where the meniscotibial and meniscocapsular fibers attach to the posterior horn medial meniscus (PHMM) [18]. Due to the complex anatomy of this area a precise preoperative diagnosis is always tricky. Magnetic resonance imaging (MRI) has shown to have a specificity of 94% and a sensitivity up to 84% if a 3.0 Tesla MRI is used and the acquisition is performed with the knee at 30° of flexion [29]. The MRI findings indicative of a ramp lesions are the irregularity at the posterior margin of medial meniscus and the interposition of synovial fluid between the PHMM and the capsule as the most sensitive findings [17, 32, 49], and recently, posteromedial tibial bone bruise has been identified as a possible diagnostic sign of these lesions [6, 13, 30]. However, the pre‐operative diagnosis of ramp lesions both coupling clinical and radiological findings is still far to be clear, and the systematic arthroscopic exploration of the posteromedial compartment remains mandatory not to overlook this injury [41]. To avoid its detrimental effects a variety of treatment has been advocated, and even though “nontreatment” of stable ramp lesions has been proposed with good clinical results, surgical repair remains the main option [3, 5, 46]. To date, there is no gold standard procedure for repairing these injuries and the surgeon should be aware of the different surgical options to apply them in the most appropriate case. Therefore, the purpose of this study was to provide a comprehensive overview of all the surgical techniques published in the literature for repairing meniscal ramp lesions focusing on the technical aspects and the pros and cons of each procedure.

## MATERIALS AND METHODS

A literature review was performed using a search strategy design to collect articles regarding surgical repair of meniscal ramp lesions. The inclusion criteria adopted were as follows: (1) studies with at least one surgical technique for repairing meniscal ramp lesions; (2) articles published within the last 20 years; (3) studies published in English; and (4) studies involving human species (including cadaveric studies). Review articles were excluded from the search.

### Article selection

The search was conducted according to the preferred reporting items for systematic reviews and meta‐analysis (PRISMA) Guidelines [36] by a reviewer on PubMed Central, Scopus, and EMBASE, for studies available until May 2023. The keywords used for initial screening were ((((meniscal Ramp injury) OR (meniscal ramp lesion)) OR (meniscal ramp)) OR (ramp lesion)) AND ((operative technique) OR (repair) OR (suture) OR (management)).

Two authors (Luis L. Urda and Nicola Pizza) independently reviewed each article's title and abstract from the literature research. The assessors were not blinded to the authors of the publications. The full text was obtained and evaluated when eligibility criteria could not be assessed from the initial screening.

## RESULTS

**Figure 1:**
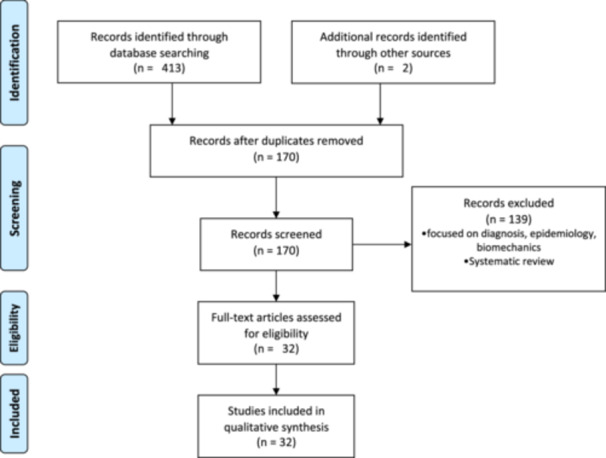
PRISMA flow chart of studies included in the review.

After the exclusion process detailed in Figure 1, a total of 32 studies were included in our current concept review. Among these articles, 30 described an all‐inside suture technique [2, 4, 8, 9, 10, 12, 16, 20, 21, 22, 23, 24, 25, 26, 27, 28, 31, 33, 35, 37, 38, 39, 41, 42, 43, 45, 47]. Thirteen of them were related to an all‐inside suture through anterior arthroscopic portals [10, 11, 14, 16, 24, 25, 26, 28, 31, 33, 37, 38, 43, 48] while eleven were related to an all‐inside suture through one posteromedial portal [4, 8, 12, 16, 21, 22, 41, 42, 43, 44, 47]. Ten of the all‐inside suture studies through a posteromedial portal described the technique using a hook suture passer device [4, 12, 16, 21, 22, 41, 42, 43, 44, 47], while one study described the technique using a scorpion suture passer [8]. Five studies were related to an all‐inside suture technique through dual posteromedial portals [2, 20, 35, 39, 45]. Three studies described a trans‐septal portal technique, involving a posterolateral trans‐septal arthroscopic portal as a viewing portal while a posteromedial arthroscopic portal was used as a working portal [8, 9, 28]. Three studies reported an inside‐out suture technique for repairing meniscal ramp lesions [12, 14, 15].

The characteristics and results of the included articles are reported in Table 1.

**Table 1:** Characteristics and results of the included studies.

Authors	Study design	No of patients	Classification	Arthroscopic diagnosis	Suture technique and device	Portal used for repair	Follow‐up (months)
Hatayama et al. [22]	Cohort study (LoE 3)	217		Transcondylar notch view	All‐inside suture with a hook suture pass device (Accu‐Pass; Smith & Nephew, London, UK).	PM	24
Sonnery‐Cottet et al. [41]	Case series (LoE 4)	302		Transcondylar notch view and PM portal	All‐inside suture with a hook suture pass (Quick‐Pass; Arthrex, Naples, FL).	PM	
Brito de Alencar Neto et al. [8]	Technical note			Transcondylar notch view	All‐inside suture with a Scorpion suture passer (Scorpion suture passer; Arthrex, Naples, FL).	PM	‐
Chen et al. [10]	Retrospective study	46	Three tear types: – Meniscotibial ligament tear. Meniscocapsular tears – Combined meniscotibial ligament and meniscocapsular tears.	Transcondylar notch view	All‐inside suture with an all inside suture device (Fast‐Fix; Smith & Nephew, Andover, MA).	AM	32
Negrín et al. [38]	Technical note			Transcondylar notch view	All‐inside suture with an all inside device (curved Fast‐Fix 360; Smith & Nephew, Andover, MA).	AM	‐
Choi et al. [11]	Technical note			Transcondylar notch view	All‐inside suture with an all inside device (Fast‐Fix 360; Smith & Nephew, Andover, MA).	AM	
Buyukdogan et al. [9]	Technical note			Transcondylar notch view and PM portal	All‐inside suture with a hook suture pass device (Depuy).	1 or 2 PM (if lesion > 2 cm)	
Siboni et al. [39]	Technical note			Transcondylar notch view and PM portal	All‐inside suture with a hook suture pass device (Spectrum; Conmed, Linvatec, Largo, FL).	2 PM	
Vadhera et al. [47]	Video‐Based Article			Transcondylar notch view	All‐inside suture with a hook suture pass device (Corkscrew Suture‐Lasso; Arhrex, Naples,FL).	PM	
Gülenç et al. [20]	Prospective study	15		Transcondylar notch view	All‐inside suture with a hook suture pass device (Quick‐Pass; Arthrex, Naples, FL).	2 PM	33
Toanen et al. [45]	Experimental cadaveric study (LoE 4)	11			All‐inside suture with a hook suture pass device (Accu‐Pass; Smith & Nephew, London, UK).	2 PM	
Thaunat et al. [43]	Case‐control study (LoE 3)	248	Thaunat et al.		All‐inside suture with a hook suture pass device (Ramp Suture‐Lasso; Arthrex, Naples, FL). An all‐inside suture with an all‐inside device (Fast‐Fix; Smith & Nephew, Andover, MA) is used for type 3 lesions and tears involving the pars intermedia.	PM In type 3 lesions, or tears involving the pars intermedia, an AM portal is used to repair the ramp lesion	24
Labarre et al. [31]	Technical note		Three tear types: – Meniscotibial ligament tear. Meniscocapsular tears – Combined meniscotibial ligament and meniscocapsular tears.	Transcondylar notch view	All‐inside suture with an all‐inside device (AIR; Stryker, Kalamazoo, MI).	AM	
Li et al. [33]	Technical note	23		Transcondylar notch view	All‐inside suture with an all‐inside device (Fast‐Fix; Smith & Nephew, Andover, MA).	AM	14
Kawada et al. [26]	Technical note			Transcondylar notch view	All‐inside suture with an all‐inside device (Fast‐Fix; Smith & Nephew, Andover, MA).	AM	
DePhillipo et al. [14]	Technical Note			Transcondylar notch view and PM portal	Inside‐out suture with an inside‐out suture device (Sharpshooter; Stryker, Kalamazoo, MI).	AM	
Keyhani et al. [27]	Prospective study (LoE IV)	128		Transcondylar notch view	All‐inside suture with a hook suture pass device (Suture Hook Lasso; Conmed, Linvatec, NY).	PM	24–47
Alomar [4]	Case Report	1	Modification of Thaunat et al.	Transcondylar notch view	All‐inside suture with a hook suture pass device.	PM	36
Choi et al. [12]	Cohort study (LoE II)	48		Transcondylar notch view	All‐inside suture with a hook suture pass device (Linvatec; Largo, FL). Inside‐out suture.	PM	35.7
Jiang et al. [24]	Retrospective study	20		Transcondylar notch view and PM portal	All‐inside suture with an all‐inside device (Omnispan; Depuy Mitek, Rayhanm MA, USA).	AM	26
Keyhani et al. [28]	Technical Note			Transcondylar notch view	All‐inside suture with a hook suture pass device (Suture‐Lasso; Arthrex, Naples, FL).	PM	
Heilpern et al. [23]	Experimental cadaveric study.	20		Transcondylar notch view	All‐inside suture with an all inside device (Fast‐Fix360 and Ultra FastFix; Smith & Nephew, Andover, MA).	AM	
Karaca et al. [25]	Case series (LoE 4)	41	Thaunat et al.	Transcondylar notch view	All‐inside suture with an all inside device (Fast‐Fix 360; Smith and Nephew, Andover, MA).	AM	37.6
Guy et al. [21]	Technical note		Thaunat et al.	Transcondylar notch view	All‐inside suture with a hook suture pass device (Arthrex, Naples, FL).	PM	
Thaunat et al. [42]	Case series (LoE 4)	132	Thaunat et al.	Transcondylar notch view	All‐inside suture with a hook suture pass device (Suture‐Lasso; Arthrex, Naples, FL).	PM	27
Abdelrazek et al. [37]	Technical note			Transcondylar notch view	All‐inside suture with an all‐inside device (Fast‐Fix 360; Smith & Nephew, Andover, MA).	AM	
Gousopoulos et al. [16]	Cohort study (LoE 3)	474		Transcondylar notch view	All‐inside suture with an all inside device (Ultra Fast‐Fix; Smith & Nephew, Andover, MA). All‐inside suture with a hook suture pass device (Quick‐Pass Lasso; Arthrex, Naples, FL).	AM PM	108.3 (All‐inside repair group). 86.6 (suture hook repair group).
Liu et al. [35]	Randomized controlled trial. (LoE 2)	91		PM portal	All‐inside suture with a hook suture pass device (Linvatec, Largo, FL).	2 PM	37.9 (surgical repair group). 40.3 (abrasion and trephination alone group).
Thaunat et al. [42]	Technical note		Thaunat et al.	Transcondylar notch view	All‐inside suture with a hook suture pass device (Suture‐Lasso; Arthrex, Naples, FL).	PM	
Ahn et al. [2]	Technical note			Transcondylar notch view	All‐inside suture with a hook suture pass device (Linvatec, Largo, FL).	2 PM	
DePhillipo et al. [15]	Cohort study (LoE 3)	100		Transcondylar notch view	Inside‐out suture with an inside‐out device (Sharpshooter; Stryker, Kalamazoo, MI).	AM	24
Yang et al. [48]	Retrospective study (LoE 3)	68		Transcondylar notch view	All‐inside suture with an all‐inside device (Fast‐Fix; Smith & Nephew, Andover, MA).	AM	24

Abbreviations: ACL, anterior cruciate ligament; ACLR, anterior cruciate ligament reconstruction; AM, anteromedial; LoE, level of evidence; PHMM, posterior horn of the medial meniscus; PL, posterolateral; PM, post eromedial.

Finally, a summary of pearls, pitfalls, advantages, and disadvantages of each technique reported can be found in Table 2.

**Table 2:** Pearls, pitfalls, advantages, and disadvantages of the surgical techniques published in the literature.

Technique	Portals and device		Pearls	Pitfalls	Advantages	Disadvantages
All Inside	Through anterior portals		1. Perform the procedure through AM and AL portals (PM portal can be eventually performed) 2. MCL and POL pie‐crusting to better visualize and access to medial compartment 3. An arthroscopic grasper through a PM portal can be employed to raise the retracted meniscotibial ligament 4. Versatile suture configuration depending on tear's pattern, size, and tissue quality 5. A strong vertical mattress suture with the first implant in the superior meniscal surface (meniscocapsular wall) and a second one on the inferior meniscal surface (meniscotibial ligament)	1. All‐inside suture with curved device may further damage the meniscal tissue 2. Not elevating the meniscotibial ligament does not ensure correct fixation 3. Pie‐crusting of MCL and POL may cause Iatrogenic damages of saphenous nerve/vein or cartilage if performed outside anatomical landmarks and without arthroscopic control	1. Correct meniscotibial ligament and meniscal fixation with satisfactory postoperative clinical results in patients with type 3 ramp lesions (43) 2. No need for an open approach 3. Biomechanically strong vertical suture 4. Easy and fast (need to be confident with all‐inside sutures)	1. More expensive than the suture hook devices 2. If an arthroscopic grasper is used, an additional posteromedial portal is needed. 3. Less accurate in determining the extent of the tear. 4. All‐inside meniscal repair devices specific risk (anchor irritation) 5. Not direct visualization of the lesion while suturing.
	Through one PM portal	PM hook suture pass device	1. Use of transillumination to perfume the PM portal and avoid saphenous nerve/vein damages 2. Use an 18‐Gauge spinal needle above the hamstring tendons, 1 cm posterior to the medial joint line, with the knee at 90° of flexion to establish the PM portal 3. Arthroscopic cannula can be used to avoid extravasation, but can impar the handling	1. Latrogenic damages of saphenous nerve/vein performing the PM portal	1. Low‐cost technique 2. Better visualization of the posterior horn of the medial meniscus and the meniscal ramp lesions 3. Improved debridement and biologic stimulation of the lesion. 4. Reinsertion of the meniscocapsular and meniscotibial ligament	1. Additional incision. 2. Risk of a saphenous nerve or venous injury 3. Longer learning curve in establishing posteromedial portal and using suture‐hook device placing and tying sutures. 4. Increased operative time.
	Through 2 PM portals	Using a suture hook pass device	1. Use a needle and transillumination to identify the entry of the viewing portal 2. The portal should be placed proximally to the posteromedial synovial fold 3. Use a switching stick to facilitate introducing the trocar into the viewing portal. 4. With a flexion‐extension movement of the knee, evaluate the adherence of the posterior capsule to the posterior horn of the medial meniscus 5. Use a needle and repeat the portal placement procedure to create the more distal working portal at the height of the joint line 6. A biologic augmentation with an abrasion of the meniscal under‐surface bone with a burr can be performed (41)	1. Too close PM limits the maneuverability of instruments	1. Complete visualization of the ramp lesion from its medial to its central border 2. Both hands of the surgeon are working in line for the repair procedure, facilitating triangulation 3. Lower rate of cartilage damage by the suture hook because of probe assistance	1. Additional posteromedial incision. 2. Additional risk of iatrogenic injury to saphenous structures. 3. Increased operative time. 4. Not applicable in small knees
	Through one PM portal with a trans‐septal PL vision portal	Using a suture hook pass device	1. Establish the PM portal 2. Establish the PL portal above the biceps tendon 3. An arthroscopic cannula in the PM portal can help not to lose the portal reducing operative time 4. Always work with the instruments oriented anteriorly to avoid popliteal bundle damages	1. Too low PL portal do not help the visualization of PM compartment 2. When passing the posterior septum care must be given in not damage popliteal bundle	1. Working on the posteromedial portal with 45° to the left/right suture passers allows vertical mattress suture placement	1. Risk of neurovascular injury during posterior portal placements and trans‐septal portal establishment. 2. Additional posteromedial and posterolateral incision. 3. Long learning curve. 4. Increased operative time.
Inside Out		Using an inside‐out suture device	1. Inside‐out meniscal repair with vertical mattress sutures for a strong repair construct	1. Latrogenic damages of saphenous nerve/vein performing the PM incision	1. More versatility in suture orientation	1. Technically demanding, need for a PM open approach. 2. Additional risk of iatrogenic injury to saphenous structures. 3. Increased operative time.

Abbreviations: AM, anteromedial; MCL, medial collateral ligament; PL, posterolateral; PM, posteromedial.

## DISCUSSION

The objective of the present study was to review the surgical techniques reported in the literature for the repair of meniscal ramp lesions. Several techniques have been described for repairing these lesions, with the most frequently utilized method being all‐inside sutures. The all‐inside techniques can be further classified based on their approach, including anterior arthroscopic portals, a posteromedial portal, dual posteromedial portals, or a trans‐septal portal suture repair technique. Additionally, inside‐out techniques have been documented for repairing meniscal ramp lesions.

Ramp lesions managed without repair deserve a special mention.

### Debridement or abrasion without repair

Ramp lesion with a length less than 2 cm or without excessive anterior translation of the PHMM upon probing from the anteromedial portal, are defined stable and can be managed with only a debridement. This involves placing the arthroscope through the transcondylar space and refreshing the two edges of the tear [48]. Another technique involves abrasion and trephination of the torn meniscus through the posteromedial portal [35].

Hatayama et al. [22] conducted a study comparing the postoperative outcomes for ramp lesions between 25 patients treated with all‐inside repair through the posteromedial portal and 25 patients whose ramp lesions were left in situ without repair during ACL reconstruction. The healing rate of ramp lesions showed a significant difference between the nonrepaired group and the repaired group (60% vs. 100%, *p* = 0.001). Moreover, two knees in the non‐repaired group required medial meniscectomy for subsequent bucket‐handle tear one and five years after ACL reconstruction, whereas no knees in the repaired group required subsequent meniscal surgery.

In contrast, Yang et al., [48] compared the efficacy of arthroscopic refreshing treatment of stable meniscus ramp injuries with the all‐inside suture with FastFix device in cases of concomitant ACL tear. The authors reported similar clinical results in terms of the Lysholm, IKDC, knee range of motion, and recovery of objective symptoms. MRI findings at 12 months showed complete healing in 18 patients in the refreshing treatment group, as well as 21 patients in the repaired group, without significant difference. Furthermore, in a randomized controlled trial, Liu et al. [35] compared the clinical outcomes of stable ramp lesions between patients treated with surgical all‐inside suture through two posteromedial portals and patients with abrasion and trephination alone during ACL reconstruction, both groups experienced a statistically significant increase in Lysholm and IKDC score. At the final follow‐up, similar clinical outcomes between the groups in terms of subjective scores (Lysholm score and IKDC) and knee stability (pivot‐shift test, Lachman test, KT‐1000 arthrometer SSD, and KT‐1000 arthrometer grading) were also reported. Moreover, almost all patients showed complete healing at MRI at final follow‐up (38/40 patients in repair group; 29/33 in abrasion and trephination group).

### All‐inside techniques

#### Sutures through anterior arthroscopic portals

**Figure 2:**
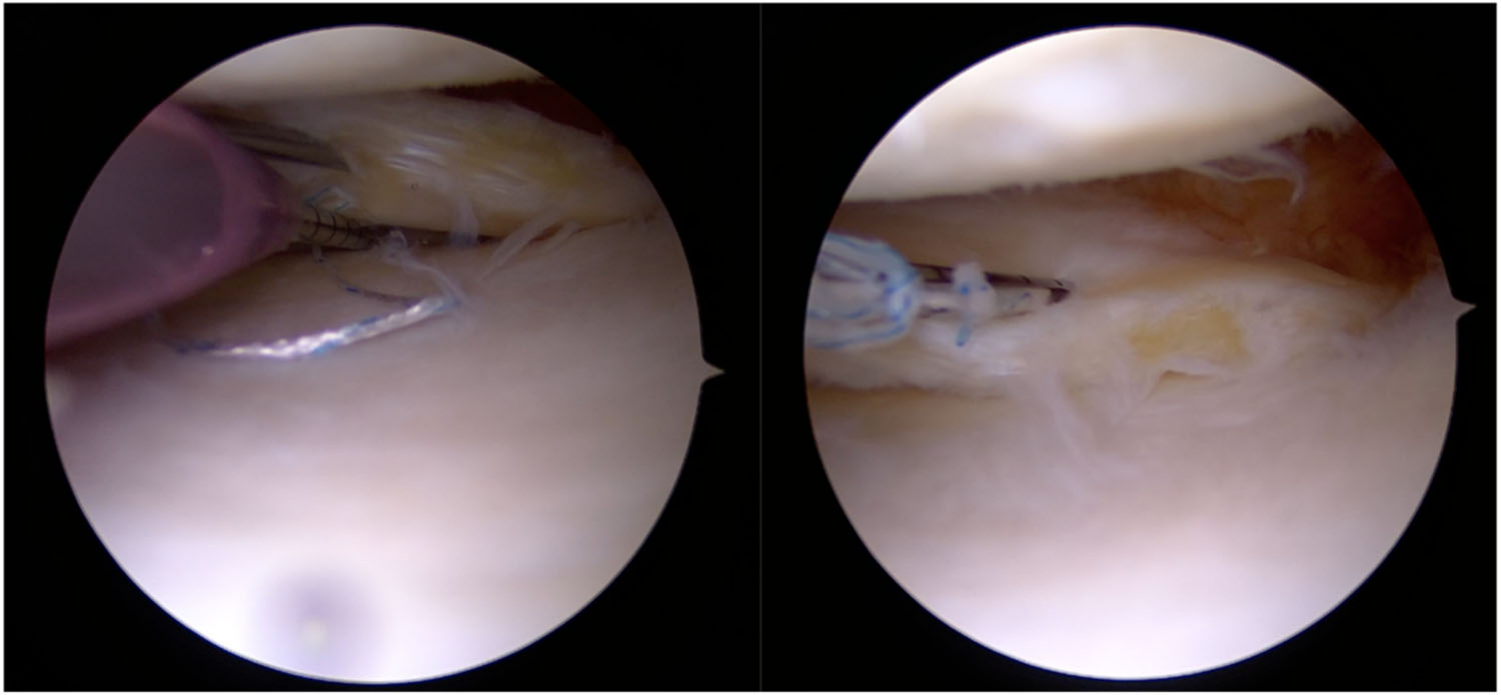
All‐Inside suture through anterior arthroscopic portals.

Among the all‐inside techniques, the repair of meniscal ramp lesions through anterior standard arthroscopic portals have been extensively discussed. Li et al. [33] introduced the Fast‐Fix (Smith & Nephew) technique for repairing meniscal ramp injuries. Their recommended approach involved arthroscopic visualization of the posteromedial compartment by inserting the arthroscope through an anterolateral portal and advancing it through the intercondylar notch beneath the posterior cruciate ligament (PCL). The surgical procedure is performed introducing the Fast‐Fix device through the anteromedial portal with the assistance of a split cannula to ensure its safe insertion. The ramp lesion is then fixed by inserting the first implant into the joint capsule beneath the meniscus and the second implant catching the periphery of the meniscus and the capsule (Figure 2).

**Figure 3:**
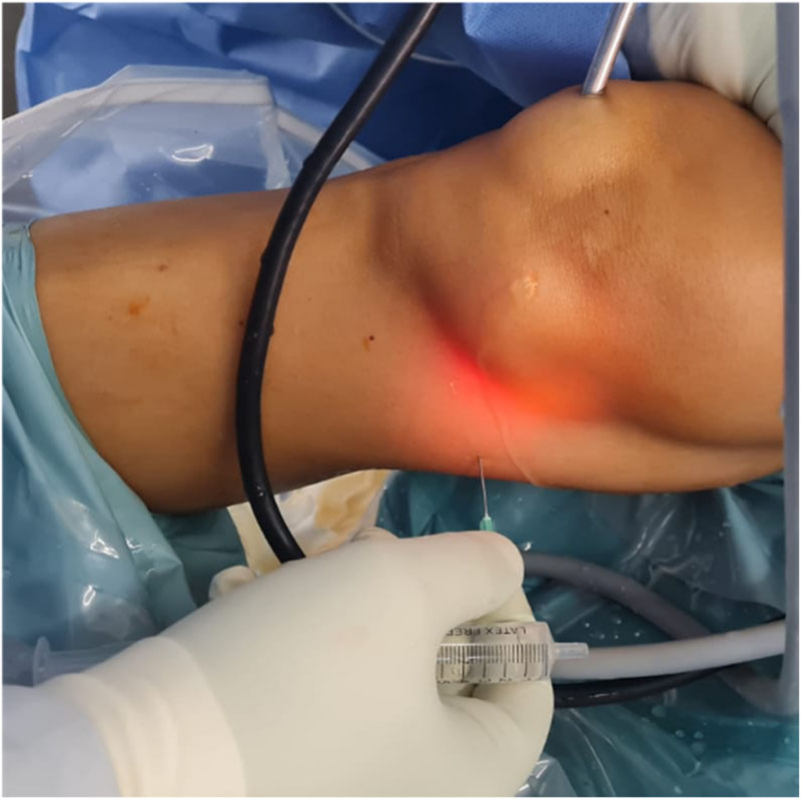
Pie‐crusting technique performed with an intramuscular needle.

In some cases, visualizing PMHH can be challenging due to a narrow medial compartment. To address this, a medial collateral ligament (MCL) release using a pie‐crusting technique has been employed to facilitate visualization and treatment of meniscal ramp lesions [11, 26, 31]. This technique involves needling the superficial MCL with an 18‐gauge needle while the knee is in a valgus position at 15–20° of flexion. The needle is inserted 1 cm distal and in the posterior third of the joint line (Figure 3). However, it is important to note that MCL release carries the risk of iatrogenic complete MCL rupture and mostly saphenous vein and nerve damage if performed too posteriorly.

**Figure 4:**
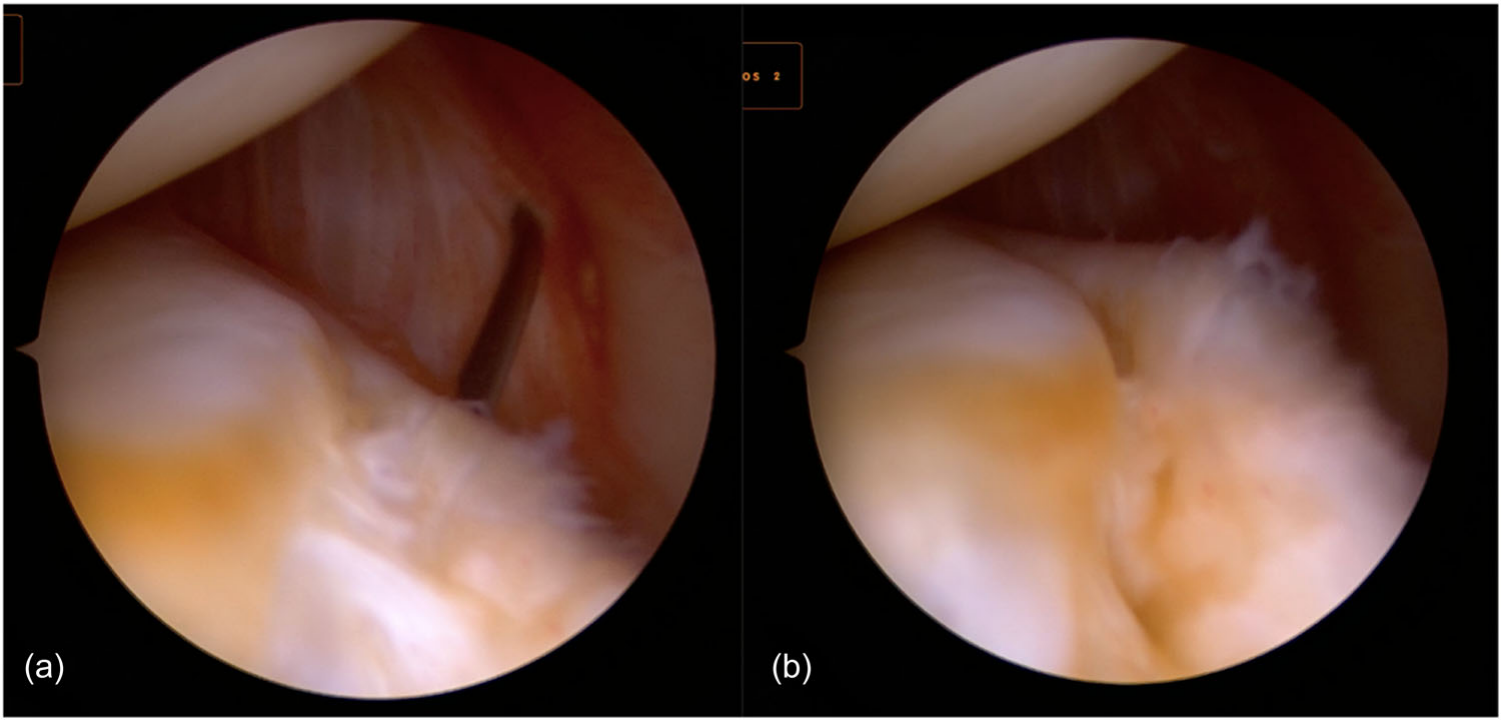
(a) Elevating the meniscotibial ligament (MTL) with an 18‐gauge needle introduced through the posteromedial compartment. (b) Good tensioning of the MTL can be observed.

One concern with all‐inside suture through anterior portals is the re‐tension that can be achieved on the MTL. Negrín et al. [38] suggested that elevating the MTL using an arthroscopic grasper inserted through a posteromedial portal (or an 18‐gauge needle, as in authors experience) (Figure 4) and then deploying the all‐inside suture the proper tension of the MTL can be achieved. However, this approach has the disadvantage of requiring an additional posteromedial portal, which carries a risk of neurovascular damage.

Suturing meniscal ramp lesions through the AM portals offers several advantages, including an effective and safe technique that is less time‐consuming and has a short learning curve. However, there are also disadvantages, such as higher costs due to the use of devices, decreased accuracy in determining the extent of the tear, and limited usefulness for MTL lesion.

The all‐inside suture through anterior portals have shown promising clinical and functional outcomes in isolated meniscal ramp lesions [24, 25], and with concomitant ACL reconstruction [10]. Jiang et al. [24] evaluated 20 isolated meniscal ramp lesions showing its healing 3 months follow‐up and a significant improvement of the Lysholm score at 2 years follow‐up.

Also, Karaca et al. [25] showed a significant improvement in the postoperative Lysholm and IKDC scores, meniscal healing and improvement of the anterior laxity evaluating 41 patients with type 3 meniscal ramp lesion (combination of partial inferior PHMM tear and MTL tear) at 3 years follow‐up. These successful results have even been seen by Chen et al. [10] which confirmed the meniscal healing of 46 ramp suture associated with concomitant ACL reconstruction with a second arthroscopic look at 3 years follow‐up. On the other side, Thaunat et al. investigating the risk factors for failures of ramp repair identified a more than five‐fold risk of failure if the repair is performed with all‐inside sutures through anterior arthroscopic portals compared to suture hook repair technique. This finding seems in accordance with, Gousopoulos et al., [16] who compared the secondary meniscectomy rates of suture hook repair through a posteromedial portal and all‐inside repair through anterior portals at a mean follow‐up of 8 years months which was of 19% for the first one and 30% for the second one.

#### Sutures through a posteromedial portal

The all‐inside techniques using a posteromedial portal can be achieved with two different devices. Brito de Alencar Neto et al. [8] recommended the use of a knee scorpion suture passer through a posteromedial arthroscopic portal because it is an easier and faster technique compared to the suture hook passer devices. However, this technique has similar disadvantages to other all‐inside techniques through a posteromedial portal.

**Figure 5:**
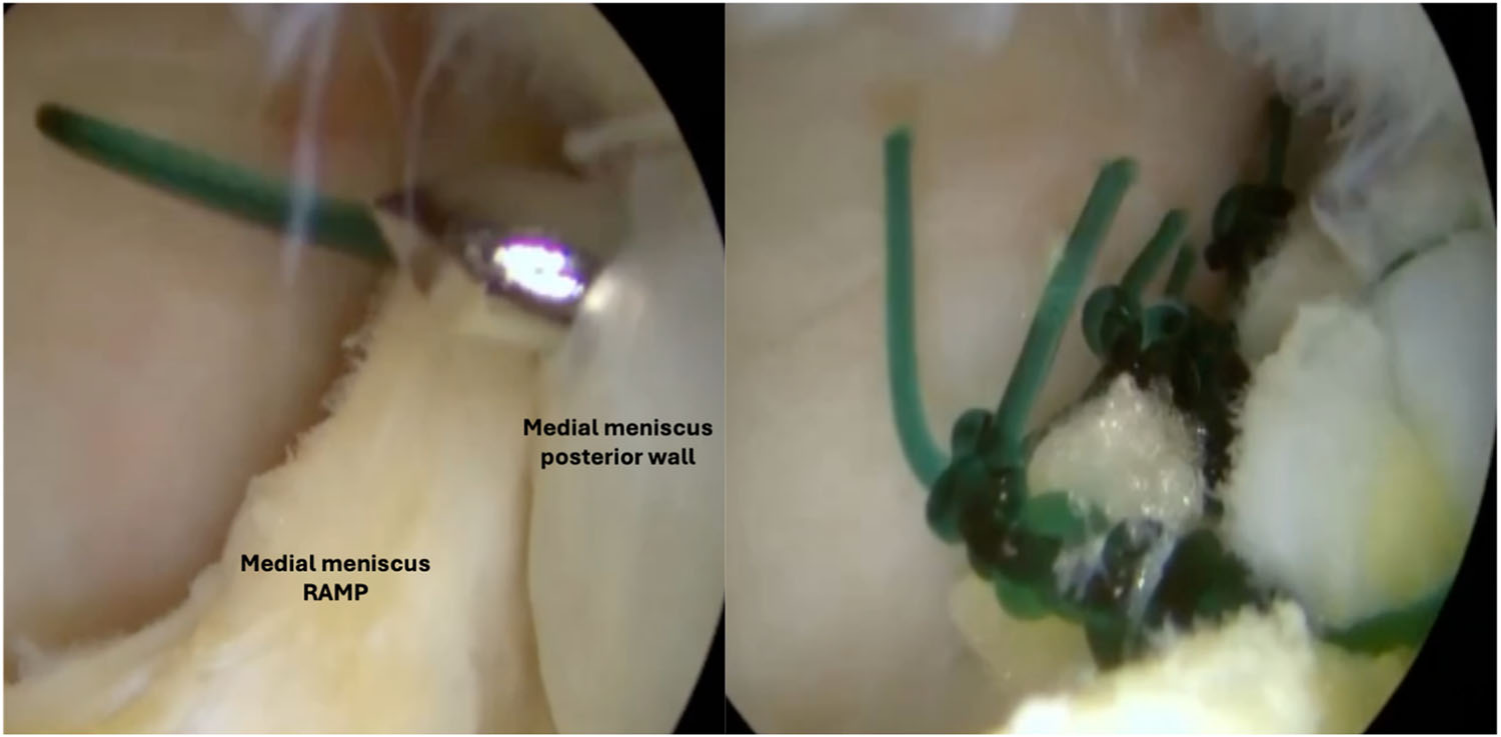
All‐Inside suture through a posteromedial portal using a hook suture passer device loaded with absorbable suture.

On the other hand, the repair of meniscal ramp lesions using a hook suture passer device has been widely described. Sonnery‐Cottet et al. [41] moreover, outlined a systematic surgical exploration in four steps to avoid missing meniscal ramp lesions. The arthroscopic exploration of the posteromedial aspect through a trans‐notch visualization is performed and a posteromedial portal is created. Finally, the meniscal repair procedure is performed using a suture hook device. Thaunat et al. [42] recommend creating the posteromedial portal 1 cm posterior to the medial femorotibial joint line with transillumination guiding the portal location. The lesion is then evaluated and debrided. A left curved hook device is used for the right knee and vice versa. Typically, the suture hook passer is loaded with an No. 0 or 1 absorbable monofilament suture, although a nonabsorbable monofilament suture can be used [41, 47]. To protect the medial condyle from iatrogenic cartilage damages, the tibia is internally rotated. The tip of the suture hook first penetrates the meniscocapsular ligament and MTL, and then the PHMM. The free end of the suture is extracted and collected through the posteromedial portal using a grasper. A self‐locking sliding knot is tied using a knot‐pusher and then cut [4, 21, 42, 47] (Figure 5).

The advantages of this technique include its low cost, improved debridement of the lesion and better visualization of the PHMM and the meniscal ramp lesion. However, there are some disadvantages, such as the need for an additional portal which increases the risk of saphenous nerve or venous injury, a significant learning curve in creating the posteromedial portal and placing and tying sutures, as well as an increased operative time.

In a case series study, Thaunat et al. [44] evaluated the results of all‐inside suture repair with a suture hook device through a posteromedial portal on 132 meniscal ramp lesions associated with an ACL rupture at 2 years follow‐up. If the IKDC score significantly improved from the pre‐operative to the final follow‐up, the authors reported a slightly lower Tegner score with an overall failure rate of 9%. Thus, concluding that arthroscopic meniscal repair of ramp lesions during ACL reconstruction with a suture hook device through a posteromedial portal provided a high rate of meniscus healing at the level of the tear and appeared to be safe and effective in this group of patients. As mentioned earlier, Hatayama et al., [22] compared the results between meniscal ramp lesion repair and non‐repair with concomitant ACL rupture demonstrating a significantly better healing at MRI in the repair group. Additionally, anterior laxity in the knees with an unhealed ramp lesion was significantly higher.

When it comes to comparing this technique to the all‐inside suture through anterior arthroscopic portals controversial results are presents in literature. As mentioned before, some authors reported a higher secondary meniscectomy rate when comparing all‐inside suture from AM portals with the suture hook technique [32, 47]. However, in a prospective cohort study at minimum 2 years follow‐up, Choi et al., [12] did not report a significant difference in meniscal healing on MRI nor in laxity measurement with the Lachman test and KT‐arthrometer, or in the functional evaluation with Lysholm and Tegner scores between the two groups.

#### Sutures through two posteromedial portals

Among the all‐inside sutures through two posteromedial portals, Ahn et al. [2] introduced this technique. The posteromedial compartment is approached by passing the arthroscope from the anterolateral portal through the intercondylar notch. Afterwards, a standard posteromedial portal is created under direct arthroscopic visualization. The posterior compartment is examined with a probe and by switching the arthroscope to the posteromedial portal, the lesion is completely visualized. The second posteromedial portal is marked 1 cm superior to the previous standard posteromedial portal. The entry point is localized with an 18‐gauge needle while viewing from inside. Then the second posteromedial portal is made, which is bigger than the standard. A cannula is inserted through this second portal.

A suture hook device is inserted through the standard posteromedial portal, and the suturing is performed similarly to the one‐posteromedial approach mentioned above.

To facilitate the suture, a probe is introduced through the second posteromedial portal and holds the central fragment down to the tibial surface, aiding in the suturing.

Siboni et al. [39] and Toanen et al. [45] introduced a modification of this technique. The posteromedial working portal is performed 1 cm anterior to the site of the standard posteromedial portal, and the accessory posteromedial viewing portal is established 3–4 cm proximally and slightly anterior to the posteromedial working portal. Both are established under transillumination to visualize and avoid iatrogenic injuries.

Thus, this technique allows for a better visualization of the ramp lesion, with easier triangulation if an accessory posteromedial viewing portal is established. Additional advantages include a lower rate of cartilage damage by the suture hook due to probe assistance and the use of vertically oriented anatomic sutures. Disadvantages include the two additional posteromedial incisions with an increased risk of iatrogenic injury to saphenous structures and an increased operative time.

Gülenç et al. [20] conducted a prospective study to evaluated the results of surgical repair of 15 ramp lesions using this technique with a suture hook device with significant clinical improvement at 8 months follow‐up. However, as we mentioned above, Liu et al. [35] compared the clinical outcomes of stable ramp lesions between patients treated with surgical all‐inside suture through two posteromedial portals as described by Ahn et al., [2] or with abrasion and trephination alone during ACL reconstruction, reporting similar clinical outcomes in terms of subjective scores (Lysholm score and IKDC), knee stability (pivot‐shift test, Lachman test, KT‐1000 arthrometer SSD, and KT‐1000 arthrometer grading), and meniscal healing status.

#### Sutures through a trans‐septal portal technique

Ahn [1] described this technique to visualize and facilitate arthroscopic procedures in the posterior compartment both medial and lateral. Buyukdogan et al. [9] and Keyhani et al. [28] performed this technique for the repair of meniscal ramp lesions. A standard posteromedial portal is created under transillumination to avoid injury to the saphenous nerve or vein. To establish the posterolateral portal, the lateral collateral ligament, and long head of the biceps femoris must first be identified. Care must be taken to remain anterior to the long head of biceps femoris to minimize the risk of injury to the common peroneal nerve. This portal is also created under transillumination, followed by the insertion of a long arthroscopic cannula.

The following step is the aperture using a blunt instrument of the posterior septum to create the connection between the posteromedial and posterolateral compartments. The point of penetration in the septum should be just posterior to the PCL at the midpoint in a vertical position. If the aperture is too high, it may cause bleeding from the arterial branches of the middle geniculate artery, while if too low, there is a risk of injury to the popliteal vessels. Moreover, the aperture through the septum should be directed from posterior to anterior, again to avoid popliteal neurovascular damages while taking care of the PCL or the lateral femoral condyle cartilage. After having established the trans‐septal aperture, the arthroscope can be switched to the posterolateral portal allowing a complete visualization of the meniscal ramp lesion. The suture is then performed using a suture hook device inserted through the posteromedial portal, as mentioned above.

Keyhani et al. [28] recommended a three‐step augmentation procedure to achieve the best results in meniscal ramp repair. The first step involves thorough debridement of the fibrotic tissue at the posterior meniscosynovial junction. The second step involves using a meniscal rasp introduced through the posteromedial portal to abrasion both the synovial and meniscal sides of the tear. Finally, for in situ clot formation, an arthroscopic burr is introduced through the posteromedial portal to completely abrade the bony rim of the posteromedial tibial plateau until a bleeding subchondral cancellous bone is reached.

The trans‐septal portal technique offers several advantages, such as the possibility to explore the entire periphery of the PHMM to define the exact borders of the ramp lesion without the need for a rotational manoeuvre as well as to place vertical mattress suture with the suture hook passers.

However, some disadvantages have been reported, such as the need to establish the posterolateral portal with the above‐mentioned risks, a long learning curve, and an increased operative time.

Regarding the results of such technique, Keyhani et al. [27] analyzed a consecutive series of 128 cases performed in association with an ACL reconstruction. At a minimum follow‐up of 2 years the Lysholm and the IKDC scores showed significant improvement with respect to the pre‐op without any case of operative‐related complications.

### Inside‐out technique

**Figure 6:**
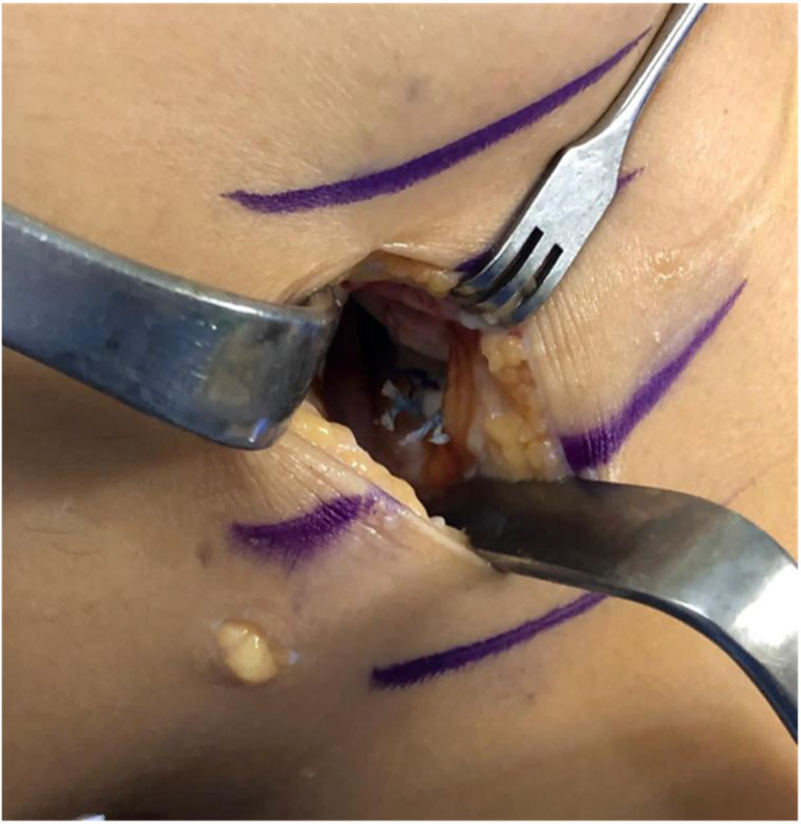
Posteromedial incision for inside‐out suture.

The inside‐out technique has been considered the gold standard for arthroscopic repair since its introduction in the 1980s. However, there is a lack of literature focused on the repair of meniscal ramp lesions using the inside‐out suture technique. DePhillipo et al. [14] described the inside‐out repair of meniscal ramp lesions. Initially, the arthroscope is introduced through the anterolateral portal and advanced through the intercondylar notch to inspect the posteromedial compartment. Once a ramp lesion is identified, a medial approach is performed. An incision of approximately 4 cm in length is made posterior to MCL, two‐thirds distal to the joint line and one‐third proximal. The approach is carried out with the knee in flexion. The anterior sartorius fascia is identified and dissected, followed by retraction of the pes anserinus to protect the saphenous nerve. The medial gastrocnemius head is then dissected off the capsule using blunt dissection. Maintaining the knee in 70°–90° of flexion helps to relax the hamstring and gastrocnemius, improving visualization and retrieval of the needle coming out from the joint for suture. The inside‐out meniscal repair is performed by introducing an inside‐out device through the anterolateral portal. The first needle is passed through the meniscus, and the second needle is passed through the adjacent capsule to create a vertical or oblique suture. The knee is flexed to 10°–20° during needle advancement, and after the passage, the knee is flexed to 70°–90° to facilitate the needle retrieval. The knot is tied, and other stitches can be added as described. The main advantage offered by this technique is the possibility to change the suture fashion according to the lesion pattern. However, the need for an open approach carries an increased risk of iatrogenic injury to saphenous vessel and nerve. Nevertheless, in cases where a medial incision is performed for other reasons such as an MCL procedure, the inside‐out technique can be taking into account (Figure 6).

When this technique is compared to the all‐inside technique through a suture‐hook device, Choi et al., [12] in a prospective cohort study detected an increased pivot‐shift in patients with the inside‐out suture but without differences in the Lachman test and KT‐1000 arthrometer, nor differences in meniscal healing rate based on MRI.

### Limitations

The present review has some limitations. First, the heterogeneity of the studies included which cannot give the possibility to drive solid conclusions on the clinical outcomes reported. However, it must be remembered that the main objective of the study was to revise all the available techniques presented in literature, without a specific focus on the clinical outcomes of such techniques which has been reported to give a comprehensive view on the described technique. Secondly the possibility of article selection bias which cannot be avoided a priori and may have affected the validity and generalizability of the present review. However, the authors strictly adhered to the PRISMA guidelines [36] and included both prospective and retrospective studies to provide the most comprehensive overview of this specific topic.

The relevance of the present study relies on the comprehensive revision of the surgical technique that have been described in the literature for the repair of meniscal ramp lesions of which the surgeon should be aware and should be part of its armamentarium to apply it to the most suitable situation.

## CONCLUSION

Meniscal ramp lesions can be managed using various approaches, each of this with its specific advantages and disadvantages. Debridement alone may be sufficient for small stable meniscal ramp lesions but, for tears in the menisco‐capsular junction that affect the stability of the medial meniscus, it seems reasonable to repair it, even though the clinical results available in literature are contrasting. All‐inside sutures through anterior portals seems to be an effective solution for meniscal ramp lesions with MTL tears. All‐inside sutures through posteromedial portals are particularly useful for large meniscal ramp lesions, in which an inside‐out suture can also be performed. Further research is required to determine the optimal technique that can be considered as the gold standard and can provide the better results.

## AUTHOR CONTRIBUTIONS

*Idea for the article*: Nicola Pizza and Simone Perelli. *Literature search and data analysis*: Luis L. Urda and Nicola Pizza. *Manuscript draft*: Nicola Pizza, Simone Perelli, Francisco S. Sanchez, and Maximiliano Ibañez. *Critical revision of the manuscript*: Stefano Zaffagnini and Juan C. Monllau.

## CONFLICT OF INTEREST STATEMENT

Prof. Stefano Zaffagnini is Editor in Chief of Journal of Experimental Orthopedics (JEO). The other authors declare no conflict of interest.

## ETHICS STATEMENT

Not applicable.

## Data Availability

Data sharing is not applicable to this article as no datasets were generated or analyzed during the current study.

## References

[1] Ahn, J.H. & Ha, C.W. (2000) Posterior trans‐septal portal for arthroscopic surgery of the knee joint. Arthroscopy: The Journal of Arthroscopic & Related Surgery, 16(7), 774–779. Available from: 10.1053/jars.2000.7681 11027767

[2] Ahn, J.H. , Kim, S.‐H. , Yoo, J.C. & Wang, J.H. (2004) All‐inside suture technique using two posteromedial portals in a medial meniscus posterior horn tear. Arthroscopy: The Journal of Arthroscopic & Related Surgery, 20(1), 101–108. Available from: 10.1016/j.arthro.2003.11.008 14716287

[3] Albayrak, K. , Buyukkuscu, M.O. , Kurk, M.B. , Kaya, O. , Kulduk, A. & Misir, A. (2021) Leaving the stable ramp lesion unrepaired does not negatively affect clinical and functional outcomes as well as return to sports rates after ACL reconstruction. Knee Surgery, Sports Traumatology, Arthroscopy, 29(11), 3773–3781. Available from: 10.1007/s00167-020-06402-w 33452579

[4] Alomar, A.Z. (2021) Novel type of medial meniscus ramp lesion: a case report and surgical technique. Journal of Surgical Case Reports, 2021(12), rjab538. Available from: 10.1093/jscr/rjab538 34888033 PMC8652030

[5] Balazs, G.C. , Greditzer, H.G. , Wang, D. , Marom, N. , Potter, H.G. , Rodeo, S.A. et al. (2020) Non‐treatment of stable ramp lesions does not degrade clinical outcomes in the setting of primary ACL reconstruction. Knee Surgery, Sports Traumatology, Arthroscopy, 28(11), 3576–3586. Available from: 10.1007/s00167-020-06017-1 32358631

[6] Beel, W. , Mouton, C. , Tradati, D. , Nührenbörger, C. & Seil, R. (2022) Ramp lesions are six times more likely to be observed in the presence of a posterior medial tibial bone bruise in ACL‐injured patients. Knee Surgery, Sports Traumatology, Arthroscopy, 30(1), 184–191. Available from: 10.1007/s00167-021-06520-z 33661324

[7] Bollen, S.R. (2010) Posteromedial meniscocapsular injury associated with rupture of the anterior cruciate ligament: a previously unrecognised association. The Journal of Bone and Joint Surgery. British Volume, 92(2), 222–223. Available from: 10.1302/0301-620X.92B2.22974 20130312

[8] Brito de Alencar Neto, J. , Marinho de Gusmão Canuto, S. , Antônio da Silva Girão, M. , Lyra de Oliveira, R. , Costa Cavalcante, M.L. , Helito, C.P. et al. (2022) All‐inside technique for ramp lesion repair: arthroscopic suture with knee scorpion suture passer. Arthroscopy Techniques, 11(11), e2091–e2096. Available from: 10.1016/j.eats.2022.08.008 36457398 PMC9705918

[9] Buyukdogan, K. , Laidlaw, M.S. & Miller, M.D. (2017) Meniscal ramp lesion repair by a trans‐septal portal technique. Arthroscopy Techniques, 6(4), e1379–e1386. Available from: 10.1016/j.eats.2017.05.029 29354444 PMC5622595

[10] Chen, Z. , Li, W.‐P. , Yang, R. , Song, B. , Jiang, C. , Hou, J.‐Y. et al. (2018) Meniscal ramp lesion repair using the fasT‐Fix technique: Evaluating healing and patient outcomes with second‐look arthroscopy. The Journal of Knee Surgery, 31(8), 710–715. Available from: 10.1055/s-0037-1606378 28873486

[11] Choi, K.Y. , Koh, I.J. , Kim, M.S. & In, Y. (2021) Medial meniscal ramp lesion repair through anterior portals using a medial collateral ligament pie‐crusting technique. Arthroscopy Techniques, 10(4), e1073–e1077. Available from: 10.1016/j.eats.2020.12.010 33981553 PMC8085387

[12] Choi, N.‐H. , Kim, T.‐H. & Victoroff, B.N. (2009) Comparison of arthroscopic medial meniscal suture repair techniques: inside‐out versus all‐inside repair. The American Journal of Sports Medicine, 37(11), 2144–2150. Available from: 10.1177/0363546509339010 19684293

[13] DePhillipo, N.N. , Cinque, M.E. , Chahla, J. , Geeslin, A.G. , Engebretsen, L. & LaPrade, R.F. (2017) Incidence and detection of meniscal ramp lesions on magnetic resonance imaging in patients with anterior cruciate ligament reconstruction. The American Journal of Sports Medicine, 45(10), 2233–2237. Available from: 10.1177/0363546517704426 28463534

[14] DePhillipo, N.N. , Cinque, M.E. , Kennedy, N.I. , Chahla, J. , Geeslin, A.G. , Moatshe, G. et al. (2017) Inside‐out repair of meniscal ramp lesions. Arthroscopy Techniques, 6(4), e1315–e1320. Available from: 10.1016/j.eats.2017.05.011 29354435 PMC5622282

[15] DePhillipo, N.N. , Dornan, G.J. , Dekker, T.J. , Aman, Z.S. , Engebretsen, L. & LaPrade, R.F. (2020) Clinical characteristics and outcomes after primary ACL reconstruction and meniscus ramp repair. Orthopaedic Journal of Sports Medicine, 8(4), 232596712091242. Available from: 10.1177/2325967120912427 PMC7218952 32426400

[16] Gousopoulos, L. , Hopper, G.P. , Saithna, A. , Grob, C. , Levy, Y. , Haidar, I. et al. (2022) Suture hook versus all‐inside repair for longitudinal tears of the posterior horn of the medial meniscus concomitant to anterior cruciate ligament reconstruction: a matched‐pair analysis from the SANTI study group. The American Journal of Sports Medicine, 50(9), 2357–2366. Available from: 10.1177/03635465221100973 35666109

[17] Greenaway, M. , Walton, E. , Gibson, D. , Le Roux, A. , Yates, P. , Ebert, J. et al. (2020) Meniscal “ramp” lesions: surgical incidence and the development of magnetic resonance imaging diagnostic criteria. Arthroscopy, Sports Medicine, and Rehabilitation, 2(4), e309–e314. Available from: 10.1016/j.asmr.2020.03.003 32875293 PMC7451914

[18] Greif, D.N. , Baraga, M.G. , Rizzo, M.G. , Mohile, N.V. , Silva, F.D. , Fox, T. et al. (2020) MRI appearance of the different meniscal ramp lesion types, with clinical and arthroscopic correlation. Skeletal Radiology, 49(5), 677–689. Available from: 10.1007/s00256-020-03381-4 31982971

[19] Guimaraes, J.B. , Schwaiger, B.J. , Gersing, A.S. , Neumann, J. , Facchetti, L. , Li, X. et al. (2021) Meniscal ramp lesions: frequency, natural history, and the effect on knee cartilage over 2 years in subjects with anterior cruciate ligament tears. Skeletal Radiology, 50(3), 551–558. Available from: 10.1007/s00256-020-03596-5 32901305 PMC7854891

[20] Gülenç, B. , Kemah, B. , Yalçın, S. , Sayar, Ş. , Korkmaz, O. & Erdil, M. (2020) Surgical treatment of meniscal RAMP lesion. The Journal of Knee Surgery, 33(3), 255–259. Available from: 10.1055/s-0039-1677887 30849785

[21] Guy, S. , Ferreira, A. , Carrozzo, A. , Delaloye, J.‐R. , Cavaignac, E. , Vieira, T.D. et al. (2022) Isolated meniscotibial ligament rupture: the medial meniscus “belt lesion”. Arthroscopy Techniques, 11(2), e133–e138. Available from: 10.1016/j.eats.2021.09.013 35155104 PMC8821026

[22] Hatayama, K. , Terauchi, M. , Saito, K. , Takase, R. & Higuchi, H. (2020) Healing status of meniscal ramp lesion affects anterior knee stability after ACL reconstruction. Orthopaedic Journal of Sports Medicine, 8(5), 232596712091767. Available from: 10.1177/2325967120917674 PMC7222250 32426412

[23] Heilpern, G. , Stephen, J. , Ball, S. , Amis, A. & Williams, A. (2018) It is safe and effective to use all inside meniscal repair devices for posteromedial meniscal “ramp” lesions. Knee Surgery, Sports Traumatology, Arthroscopy, 26(8), 2310–2316. Available from: 10.1007/s00167-018-4976-5 29752501

[24] Jiang, J. , Ni, L. & Chen, J. (2021) Isolated meniscal ramp lesion without obvious anterior cruicate ligament rupture. Orthopaedic Surgery, 13(2), 402–407. Available from: 10.1111/os.12860 33314703 PMC7957419

[25] Karaca, M.O. , Özbek, E.A. , Ertan, M.B. , Terzi, M.M. & Akmeşe, R. (2022) Short‐term outcomes after treatment of isolated hidden meniscal ramp lesions. Orthopaedic Journal of Sports Medicine, 10(4), 23259671221085977. Available from: 10.1177/23259671221085977 35386838 PMC8977712

[26] Kawada, K. , Furumatsu, T. , Tamura, M. , Xue, H. , Higashihara, N. , Kintaka, K. et al. (2023) Effectivity of the outside‐in pie‐crusting technique and an all‐inside meniscal repair device in the repair of ramp lesions. Arthroscopy Techniques, 12(2), e273–e278. Available from: 10.1016/j.eats.2022.11.002 36879867 PMC9984773

[27] Keyhani, S. , Ahn, J.H. , Verdonk, R. , Soleymanha, M. & Abbasian, M. (2017) Arthroscopic all‐inside ramp lesion repair using the posterolateral transseptal portal view. Knee Surgery, Sports Traumatology, Arthroscopy, 25(2), 454–458. Available from: 10.1007/s00167-016-4410-9 28028568

[28] Keyhani, S. , Vaziri, A.S. , Vosoughi, F. , Verdonk, R. & Movahedinia, M. (2022) Overview of posterior knee arthroscopy in the medial meniscal repair: technical note. Journal of ISAKOS, 7(3), 33–38. Available from: 10.1016/j.jisako.2022.02.002 36178394

[29] Koo, B. , Lee, S.H. , Yun, S.J. & Song, J.G. (2020) Diagnostic performance of magnetic resonance imaging for detecting meniscal ramp lesions in patients with anterior cruciate ligament tears: a systematic review and meta‐analysis. The American Journal of Sports Medicine, 48(8), 2051–2059. Available from: 10.1177/0363546519880528 31684739

[30] Kumar, N.S. , Spencer, T. , Cote, M.P. , Arciero, R.A. & Edgar, C. (2018) Is edema at the posterior medial tibial plateau indicative of a ramp lesion? an examination of 307 patients with anterior cruciate ligament reconstruction and medial meniscal tears. Orthopaedic Journal of Sports Medicine, 6(6), 232596711878008. Available from: 10.1177/2325967118780089 PMC6077919 30090830

[31] Labarre, C. , Graveleau, N. & Bouguennec, N. (2021) Meniscal ramp lesions repair: an under‐meniscus all‐inside suture in cases of isolated meniscotibial ligament tears. Arthroscopy Techniques, 10(6), e1417–e1424. Available from: 10.1016/j.eats.2021.02.005 34258185 PMC8252850

[32] Laurens, M. , Cavaignac, E. , Fayolle, H. , Sylvie, R. , Lapègue, F. , Sans, N. et al. (2022) The accuracy of MRI for the diagnosis of ramp lesions. Skeletal Radiology, 51(3), 525–533. Available from: 10.1007/s00256-021-03858-w 34216246

[33] Li, W. , Chen, Z. , Song, B. , Yang, R. & Tan, W. (2015) The FasT‐Fix repair technique for ramp lesion of the medial meniscus. Knee Surgery & Related Research, 27(1), 56–60. Available from: 10.5792/ksrr.2015.27.1.56 25750895 PMC4349646

[34] Liu, X. , Feng, H. , Zhang, H. , Hong, L. , Wang, X.S. & Zhang, J. (2011) Arthroscopic prevalence of ramp lesion in 868 patients with anterior cruciate ligament injury. The American Journal of Sports Medicine, 39(4), 832–837. Available from: 10.1177/0363546510388933 21220541

[35] Liu, X. , Zhang, H. , Feng, H. , Hong, L. , Wang, X. & Song, G. (2017) Is it necessary to repair stable ramp lesions of the medial meniscus during anterior cruciate ligament reconstruction? a prospective randomized controlled trial. The American Journal of Sports Medicine, 45(5), 1004–1011. Available from: 10.1177/0363546516682493 28060534

[36] Moher, D. , Liberati, A. , Tetzlaff, J. & Altman, D.G. (2009) Preferred reporting items for systematic reviews and meta‐analyses: the PRISMA statement. PLoS Medicine, 6(7), e1000097. Available from: 10.1371/journal.pmed.1000097 19621072 PMC2707599

[37] Mostafa Zaky Abdelrazek, B.H. , Waly, M.R. , Abdel Aziz, M.A. & Abdel Aziz, A. (2020) Different techniques for the management of meniscal ramp lesions using standard anterior portals. Arthroscopy Techniques, 9(1), e39–e44. Available from: 10.1016/j.eats.2019.08.020 32021772 PMC6993190

[38] Negrín, R. , Reyes, N.O. , Iñiguez, M. , Pellegrini, J.J. , Wainer, M. & Duboy, J. (2018) Meniscal ramp lesion repair using an all‐inside technique. Arthroscopy Techniques, 7(3), e265–e270. Available from: 10.1016/j.eats.2017.09.001 29881699 PMC5989817

[39] Siboni, R. , Pioger, C. , Jacquet, C. , Mouton, C. , Seil, J. , Toanen, C. et al. (2022) Meniscal ramp repair: a 2‐portal posteromedial approach. Arthroscopy Techniques, 11(7), e1163–e1169. Available from: 10.1016/j.eats.2022.02.026 35936835 PMC9353068

[40] Siboni, R. , Pioger, C. , Jacquet, C. , Mouton, C. & Seil, R. (2023) Ramp lesions of the medial meniscus. Current Reviews in Musculoskeletal Medicine, 16(5), 173–181. Available from: 10.1007/s12178-023-09834-2 37014609 PMC10188848

[41] Sonnery‐Cottet, B. , Conteduca, J. , Thaunat, M. , Gunepin, F.X. & Seil, R. (2014) Hidden lesions of the posterior horn of the medial meniscus: a systematic arthroscopic exploration of the concealed portion of the knee. The American Journal of Sports Medicine, 42(4), 921–926. Available from: 10.1177/0363546514522394 24567252

[42] Thaunat, M. , Fayard, J.M. , Guimaraes, T.M. , Jan, N. , Murphy, C.G. & Sonnery‐Cottet, B. (2016) Classification and surgical repair of ramp lesions of the medial meniscus. Arthroscopy Techniques, 5(4), e871–e875. Available from: 10.1016/j.eats.2016.04.009 27709051 PMC5040630

[43] Thaunat, M. , Foissey, C. , Ingale, P. , Haidar, I. , Bauwens, P.H. , Penet, A. et al. (2022) Survival and risk factor analysis of arthroscopic ramp lesion repair during anterior cruciate ligament reconstruction. The American Journal of Sports Medicine, 50(3), 637–644. Available from: 10.1177/03635465211068524 35099318

[44] Thaunat, M. , Jan, N. , Fayard, J.M. , Kajetanek, C. , Murphy, C.G. , Pupim, B. et al. (2016) Repair of meniscal ramp lesions through a posteromedial portal during anterior cruciate ligament reconstruction: outcome study with a minimum 2‐year follow‐up. Arthroscopy: The Journal of Arthroscopic & Related Surgery, 32(11), 2269–2277. Available from: 10.1016/j.arthro.2016.02.026 27184100

[45] Toanen, C. , Sanchez, M. , Beaufils, P. & Pujol, N. (2022) Ramp lesion repair via dual posteromedial arthroscopic portals: a cadaveric feasibility study. Orthopaedics & Traumatology: Surgery & Research, 108(3), 103175. Available from: 10.1016/j.otsr.2021.103175 34906726

[46] Tuphé, P. , Foissey, C. , Unal, P. , Vieira, T.D. , Chambat, P. , Fayard, J.‐M. et al. (2022) Long‐term natural history of unrepaired stable ramp lesions: a retrospective analysis of 28 patients with a minimum follow‐up of 20 years. The American Journal of Sports Medicine, 50(12), 3273–3279. Available from: 10.1177/03635465221120058 36074027

[47] Vadhera, A.S. , Parvaresh, K. , Swindell, H.W. , Verma, N. , Gursoy, S. , Evuarherhe, A. et al. (2022) Arthroscopic all—inside repair of meniscal ramp lesions. Journal of ISAKOS, 7(4), 82–83. Available from: 10.1016/j.jisako.2022.04.004 35692122

[48] Yang, J. , Guan, K. & Wang, J.Z. (2017) Clinical study on the arthroscopic refreshing treatment of anterior cruciate ligament injury combined with stable medial meniscus ramp injury. Journal of Musculoskeletal & Neuronal Interactions, 17(2), 108–113. 28574418 PMC5492326

[49] Yeo, Y. , Ahn, J.M. , Kim, H. , Kang, Y. , Lee, E. , Lee, J.W. et al. (2018) MR evaluation of the meniscal ramp lesion in patients with anterior cruciate ligament tear. Skeletal Radiology, 47(12), 1683–1689. Available from: 10.1007/s00256-018-3007-4 29936559

